# Arthroscopy Confers Favorable Clinical Outcomes in Asian Patients with Borderline Developmental Dysplasia of the Hip

**DOI:** 10.1111/os.13839

**Published:** 2023-07-31

**Authors:** Fan Yang, Zheng Zhou, Xin Zhang, Hongjie Huang, Xiaodong Ju, Jianquan Wang

**Affiliations:** ^1^ Department of Sports Medicine Peking University Third Hospital, Institute of Sports Medicine of Peking University, Beijing Key Laboratory of Sports Injuries Beijing China; ^2^ Engineering Research Center of Sports Trauma Treatment Technology and Devices, Ministry of Education Beijing China

**Keywords:** Developmental Dysplasia of the Hip, Femoroacetabular Impingement Syndrome, Hip Arthroscopy, Patient‐Reported Outcomes

## Abstract

**Objective:**

Hip arthroscopy has recently appeared as a successful therapy in treating borderline developmental dysplasia of the hip (BDDH). This study aimed to describe the minimal 2‐year follow‐up after hip arthroscopy for patients with BDDH in the Asian population and use the combination of lateral center edge angle (LCEA) and alpha angles to detect the appearance of impingement in the BDDH population.

**Methods:**

This retrospective investigation was conducted utilizing information from arthroscopically treated BDDH patients in 2018 and 2019. The following patient‐reported outcomes were reported: modified Harris Hip Score, Hip Outcome Score‐Activities of Daily Living, International Hip Outcome Tool 12‐component form, and Visual Analog Scale. We also considered the clinical data regarding radiological measurements, intraoperative findings, interventions, complications, and subsequent surgical revisions, in the analysis of combination angles in detecting the occurrence of impingement. Patients with asymptomatic contralateral hips from traumatic hip injury (pelvic fracture) served as the control group. A 2:1 propensity score matching was based on age, sex, and BMI. A receiver operating characteristic curve (ROC) was used to identify the thresholds of combination angles and their accuracies.

**Results:**

A total of 77 hips met the inclusion and exclusion requirements. After the follow‐up period, most patients showed a considerable improvement in patient‐reported outcomes compared to their preoperative values (*P* < 0.001 for all). The overall rate of complications was 5.2%, whereas the rate of revisions was 3.9%. Increasing preoperative alpha angle age was significantly positively correlated with improving patient‐reported outcomes. The combined angle cut‐off was determined to be 80.5° (AUC, 0.858; 95% CI: 0.757–0.938; sensitivity (SE), 98.1%; specificity (SP), 74.1%; *P* < 0.001) for the occurrence of impingement in BDDH population.

**Conclusion:**

Good patient‐reported outcomes and low revision rates can be expected in the BDDH population with careful selection of patients in Asian populations. A combination angle >80.5° could be a reliable predictive factor of impingement in BDDH populations.

## Graphical abstract

The incidence of FAIS was influenced by acetabular coverage and femoral head shape. In the setting of BDDH patients, LCEA angle is usually less than 25°, and the currently accepted cam impingement range of 50°–55° is inconvincible. Combination of LCEA and alpha angles might help to detect the appearance of impingement in the BDDH population.

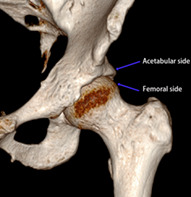

## Introduction

Hip arthroscopic surgery to repair femoroacetabular impingement syndrome (FAIS) has become a frequent operation and could be considered to have the same long‐term efficacy as open surgery.^1^ The number of people who underwent hip arthroscopy surgery in the United States increased by 85.0% between 2011 and 2018.^2^ Hip arthroscopy has also recently appeared as a successful therapy in treating acetabular dysplasia.^3^ Acetabular dysplasia may be mild, moderate, or severe depending on the lateral center edge angle (LCEA). Borderline developmental dysplasia of the hip (BDDH) delineates an intermediary acetabular coverage pattern that lies between the conventional acetabular dysplasia and normal coverage. BDDH is commonly defined as a lateral LCEA between 18° and 25°^4^ and has been associated with symptoms. BDDH is an increasingly acknowledged factor that predisposes individuals to microinstability and hip labral tears.^5^ Hip arthroscopy is increasingly used as a treatment option for patients with FAIS and BDDH.^6^, ^7^ The arthroscopy management of BDDH remains controversial within the hip preservation field. Previous research has shown that BDDH patients can also achieve clinically significant outcomes in short‐term and midterm follow‐up after arthroscopic treatment compared with normal acetabular coverage FAIS patients.^6^, ^8^ However, the outcomes may be affected by certain risk factors, including broken Shenton line, older age, excessive femoral anteversion, and Tönnis angle >15°.^7^, ^9^

Hip impingement occurs during flexion and internal rotation when an improperly developed femoral head–neck junction contacts the acetabular margin. Therefore, there are two forms of FAIS, and they can be separated by whether the impingement is due to the femur (cam deformity) or the acetabulum (pincer deformity).^10^ The cam‐type impingement was usually described as an alpha angle greater than 55°,^11^ while the pincer‐type impingement was identified as an LCEA angle >40°.^12^ The diagnosis of BDDH often appears to follow cam impingement.^7^ However, defining the cam impingement with an alpha angle >55° was nonpersuasive in the setting of the BDDH population. Impingement symptoms and clinical signs may be absent in BDDH populations with an angle >55° alone. Nevertheless, following the correction of under‐coverage of the acetabulum, impingement may arise. Multiple studies have reported a high incidence of postoperative impingement following periacetabular osteotomy (PAO) in both BDDH and DDH hips, ranging from 22.4% to 47.8%.^13^, ^14^ As FAIS incidence was influenced by acetabular covering and femoral head shape, the combination angle of the LCEA angle and the alpha angle may be more useful in detecting the occurrence of impingement in the BDDH population.

Herein, we aimed to document the minimal 2‐year follow‐up after hip arthroscopy among BDDH patients in the Asian population and use the combination angle of LCEA and the alpha angles to detect the occurrence of impingement in the BDDH population. We hypothesized that (1) hip arthroscopic treatment for BDDH would be effective in Asian patients with cautious surgical indications and (2) the occurrence of FAI in in the BDDH population may be affected by both acetabular coverage and femoral head shape.

## Methods

### Patient Selection

This study was approved by the Ethics Committee of Peking University Third Hospital (No.M2019193). We used a retrospective review data for patients who underwent hip arthroscopic surgery at our hospital from January 1, 2018, to January 1, 2019. Indications for hip arthroscopy were persistent groin pain, clinical sign of impingement test, intra‐articular pathologic abnormalities detected by magnetic resonance imaging (MRI), following at least 3 months of conservative treatment failure. Inclusion criteria: primary hip surgery, 16 to 50 years of age, had a confirmed diagnosis of borderline dysplasia (defined as having an LCEA between 20° and 25°), had positive impingement test and then underwent cam osteoplasty. Exclusion criteria: revision hip surgery, Tönnis angle >15°, fractured Shenton line, osteoarthritis (Tönnis grade ≥2), and incomplete radiographs and medical records.

In order to use the combination angle of LCEA and alpha angle detecting BDDH population with FAIS, patients with a traumatic hip injury (pelvic fracture) were reviewed during the same period as the control group. These patients underwent pelvic anteroposterior (AP) and hip computed tomography (CT). Their asymptomatic contralateral hips served as the control group. Inclusion criteria: 16 to 50 years of age, LCEA angle between 20° and 25°, had no history of hip joint pain and surgery, had a negative hip impingement test. In the results of potential confounding variables, the study and the control groups underwent a propensity score (2: 1) matching procedure, in which they were matched in age, sex, and BMI.

### Imaging Evaluation

**Figure 1:**
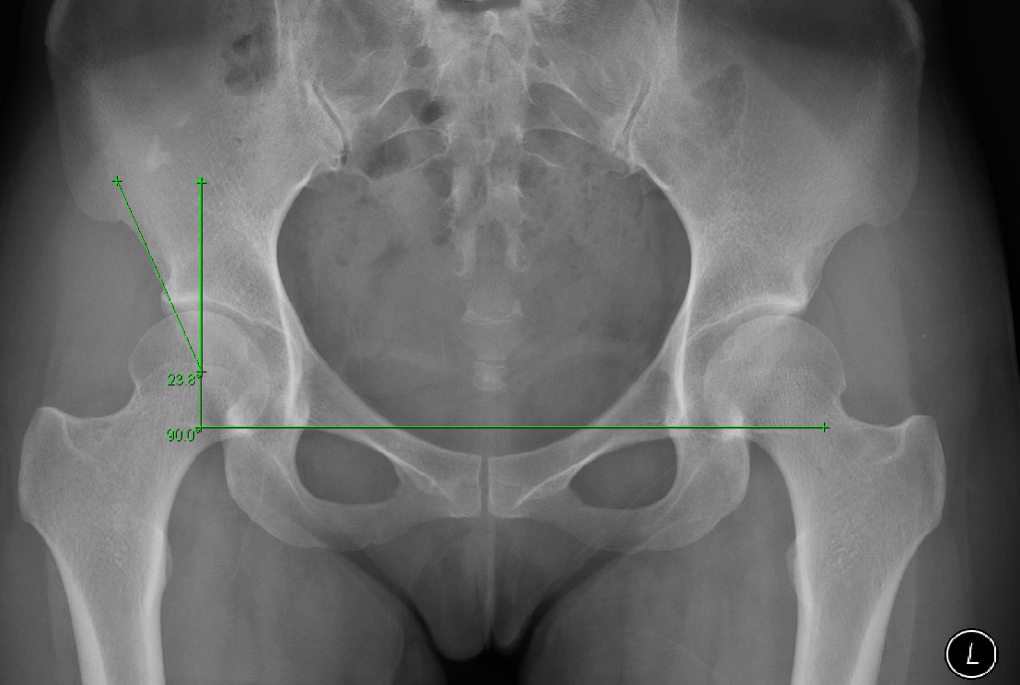
Lateral center edge angle (LCEA) angle measurement. A vertical line through the center of the femoral head was identified perpendicular to connecting line of the distal pelvic teardrops' borders; another line was drawn from the center of the femoral head to the outer lateral edge of the sourcil.

**Figure 2:**
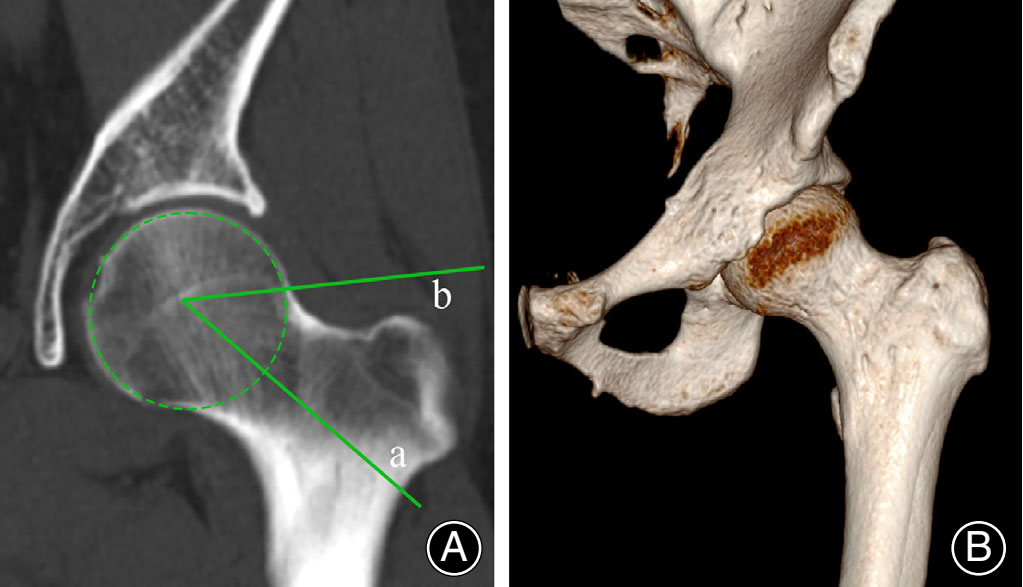
Alpha angle measurement. (A) One line (a) was drawn from the center of the femoral head down to the center of the femoral neck; another line (b) was then drawn connecting the center of the femoral head to the point of the circle where the head goes out of round. (B) 3D‐reconstructed image.

The patients received unilateral hip MRI, CT, and AP pelvis radiography. A radiologist used a PACS to record the results of his radiographic measurements (PACS; GE Healthcare). Conventional AP pelvic radiographs were utilized to calculate the tönnis and the LCEA angle. LCEA angle was formed by two lines: a vertical line extending through the middle of the femoral head was identified perpendicular to the connecting line of the distal pelvic teardrops' borders. A second line was drawn from the center of the femoral head to the outer lateral edge of the sourcil (Figure 1). LCEA measurement between 20° and 25° was interpreted as BDDH.^15^ The alpha angle was calculated using coronal CT sequences at 1 o'clock. for the two groups. Around the femoral head, an optimal‐fit circle was drawn. A line was drawn from the center of the femoral head to the center of the neck. Then a second line was drawn from the center of the femoral head to where the head deviates from the circle (Figure 2). The angle formed by these two lines is known as the alpha angle. All radiographic measurements were carried out manually by two authors. To assess inter‐ and intra‐observer reliability for continuous data, intraclass correlation coefficients (ICCs) were computed.

### Surgical Interventions and Postoperative Rehabilitation

A senior author performed all hip arthroscopies. The patient was placed with standard hip traction in the modified supine position (Smith & Nephew). An 8–10 mm distractor provided the hip joint while protecting the perineum. First, using a 70° arthroscope, the anterolateral (AL) portal, the mid anterior (MAP), and the proximal (PMAP) portals were localized fluoroscopically. An interportal capsulotomy was performed for all participants. Labral injury and chondrolabral injury in the central compartment were remedied. The outerbridge^16^ and acetabular labrum articular disruption (ALAD)^17^ categorization systems were used to evaluate cartilage damage. The margin of the acetabulum is minimally burred to create a bleeding bone bed for labral healing. The labrum is reattached after the suture anchors are positioned. After addressing pathologies in the central compartment, the arthroscope was inserted into the peripheral compartment to decompress the cam deformity with a high‐velocity burr. Related therapies are also administered if other extraarticular diseases are present, such as ischiofemoral impingement (IFI) and subspine impingement syndrome (SSI). After treatment, three to four interrupted stitches were used to close the capsular defect. As indicated previously,^18^ all patients followed a well‐standardized rehabilitation program under the direct supervision of our physiotherapist team. The first stage of the rehabilitation process primarily involved isometric contractions, passive range‐of‐motion exercises, and partial weight bearing. The subsequent phase aimed to restore a complete range of motion and sustain a typical gait. The third stage focused on regaining strength in the lower extremities and returning to regular functional activities. The fourth and final stage centered on the resumption of higher‐level pre‐injury activities. Rehabilitation lasted an average of 4 to 5 months.

### Clinical Evaluation

Patient‐reported outcomes (PROs) incorporated the Hip Outcome Score‐Activities of Daily Living (HOS‐ADL), Score‐Sports Subscale (HOS‐SSS), International Hip Outcome Tool 12‐component form (iHOT‐12), and modified Harris Hip Score (mHHS). Furthermore, a VAS was determined for patients to estimate their pain level. This scale ranged from 0 (no pain) to 10 (extreme pain). Calculations were made to determine the differences between preoperative and postoperative scores. The minimal clinically important difference (MCID) was calculated by half a standard deviation (SD) to determine a meaningful improvement in the results.^19^ The MCID thresholds for mHHS, HOS‐ADL, HOS‐SSS, and iHOT‐12, were 7.2, 7.6, 8.4, and 9.7, respectively. Based on earlier research, the Acceptable Patient Symptomatic State (PASS) was defined as 83.3 for mHHS, 88.2 for HOS‐ADL, 76.4 for HOS‐SSS, and 72.2 for iHOT‐12.^20^

### Statistical Analysis

The sample size was calculated by conducting a G*Power (version 3.1) prior investigation. A clinically significant change in the mean mHHS follow‐up is assumed to be 8.3 points.^4^ With (α: 0.05) and (β:0.2) (80% power), a sample size of 34 patients was estimated. Matching of propensity scores was carried out using R software. The algorithm had a caliper width of 0.25 SD, which corresponded to the logit of the propensity score. The Shapiro–Wilk test was utilized for normality. To evaluate differences in continuous demographic characteristics between the two groups, we used a two‐tailed unpaired Student's *t*‐test. On the contrary, a paired test was used to compare preoperative and postoperative PROs. Categorical variables were compared using the chi‐square test or Fisher's exact test. We used Spearman rank correlations to analyze the relationships between demographics and PROs' score shifts. The ROC curve measured the thresholds of the LCEA and alpha angle combination and their accuracy. The area under the curve (AUC) was also estimated. For statistical analysis, version 26 of SPSS (IBM, Armonk, NY) was utilized. All results with *P*‐values less than 0.05 were regarded as statistically significant.

## Results

### Patient Demographics

**Figure 3:**
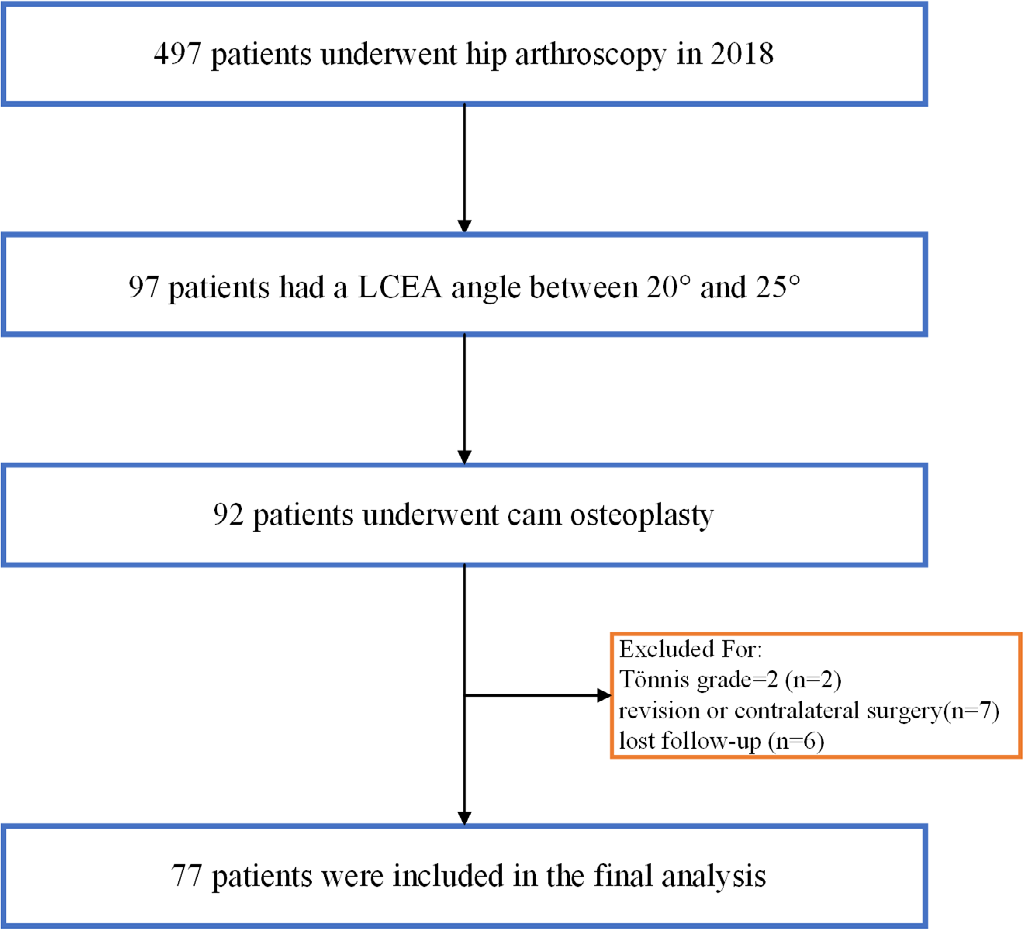
CONSORT (Consolidated Standards of Reporting Trials) diagram indicating the total patient population that met inclusion and exclusion criteria. LCEA, lateral center edge angle.

During the study period, a total of 497 hips underwent hip surgery, 97 hips had an LCEA angle between 20° and 25°, of which 92 hips underwent cam osteoplasty, two hips were excluded due to Tönnis grade 2, three hips were excluded for revision surgery, four hips were excluded for undergoing contralateral hip arthroscopy. In the course of follow‐up, six hips were lost follow‐up. A total of 77 hips met the inclusion and exclusion criteria (Figure 3). The mean age and BMI were 36.1 ± 9.8 years and 22.4 ± 3.4 years, respectively. There were 39 female (50.6%) patients in the study population. The mean LCEA and alpha angle was 23.3° ± 1.7° and 66.1° ± 6.6°, respectively. Table 1 displays the demographic information of the patient population. According to the ICC findings, both the intra‐observer and inter‐observer reliability of LCEA and alpha angle measurement were higher than 0.70 for each parameter, indicating a good reliability level (Table 2). Table 3 provides a synopsis of intraoperative findings, as well as procedures performed during arthroscopic surgery. All patients in the current study exhibited labral tears and subsequently underwent either labral repair (97.4%) or reconstruction (2.6%).

**Table 1:** Characteristics of the patients

Variables	No.
Total number of hips	77
Age, years	36.1 ± 9.8
BMI, kg/m^2^	22.4 ± 3.4
Sex, female, *n* (%)	39 (50.6)
Laterality, right, *n* (%)	45 (58.4)
Follow‐up time, months	44.5 ± 8.2
Alpha Preoperative	66.1 ± 6.6
LCEA Preoperative	23.3 ± 1.7
Joint space, mm	4.6 ± 0.9

*Note*: Values are given as mean ± SD or *n* (%).

Abbreviations: BMI, body mass index; LCEA, lateral center edge angle.

**Table 2:** Agreement of lateral center edge angle (LCEA) and alpha angle measurements

Measurement	Interobserver ICC (95% CI)	Intraobserver ICC (95% CI)
LCEA	0.874 (0.816–0.914)	0.798 (0.706–0.862)
Alpha angle	0.813 (0.726–0.872)	0.802 (0.710–0.864)

Abbreviations: CI, confidence interval; LCEA, lateral center edge angle; ICC, intraclass correlation coefficient.

**Table 3:** Intraoperative findings and procedures

Category	No. of Hips (%)
Femoral head chondral lesion: Outerbridge classification^16^	7 (9.1)
Grade 1	4 (5.2)
Grade 2	3 (3.9)
Grade 3	0 (0)
Grade 4	0 (0)
Acetabulum chondral lesion: ALAD classification^17^	70 (90.9)
Grade 1	18 (23.4)
Grade 2	11 (14.3)
Grade 3	17 (22.1)
Grade 4	24 (31.2)
Labral	
Repair	75 (97.4)
Reconstruction	2 (2.6)
Femoroplasty	77 (100)
Subspine decompression	13 (16.9)
Ischiofemoral impingement	1 (1.3)
Capsular closure	77 (100)

*Note*: Data are reported as *n* (%).

Abbreviation: ALAD, acetabular labral articular disruption.

### Clinical Outcomes

At the end of the follow‐up period, most patients showed considerable improvement in PROs compared to preoperative values. From the initial assessment to the last follow‐up, the mean score increased from 61.9 ± 11.2 to 81.6 ± 13.0 for mHHS (*P* < 0.001), 69.3 ± 13.8 to 86.2 ± 11.7 for HOS‐ADL (*P* < 0.001), 46.7 ± 17.2 to 70.1 ± 16.0 for HOS‐SSS (*P* < 0.001), 46.7 ± 17.2 to 70.1 ± 16.0 for iHOT‐12 (*P* < 0.001), and 5.7 ± 1.7 to 2.4 ± 1.8 for VAS (*P* < 0.001). The probabilities of meeting the MCID criteria and PASS are summarized in Table 4. With 83.1% of patients obtaining MCID and 57.1% achieving PASS, the mHHS exhibited the highest chance of capturing MCID and PASS performance.

**Table 4:** Patient‐reported outcome scores of included patients

Category	Preoperative	Postoperative	*t* value	*P* value	MCID (%)	PASS (%)
mHHS	61.9 ± 11.2	81.6 ± 13.0	−11.981	**<0.01**	83.1	57.1
HOS‐ADL	69.3 ± 13.8	86.2 ± 11.7	−9.785	**<0.01**	70.1	61.0
HOS‐SSS	46.7 ± 17.2	70.1 ± 16.0	−11.967	**<0.01**	81.8	46.8
iHOT‐12	45.5 ± 13.0	69.5 ± 18.2	−10.787	**<0.01**	77.9	55.8

*NOTE*: Values are given as mean ± SD or percentages.

Abbreviations: mHHS, Modified Harris Hip Score; HOS‐ADL, hip outcome scored‐activities of daily living; HOS‐SSS, hip outcome scored‐sports subscale; iHOT‐12, international hip outcome tool 12‐component form; MCID, minimum clinically important difference; PASS, patient acceptable symptomatic state.

The complication rate was 5.2%; four patients with femoral cutaneous nerve palsy spontaneously recovered. There were no additional complications reported. A total of three patients (3.9%) underwent a revision hip arthroscopy at the final follow‐up. They underwent revision arthroscopy due to subspinal impingement (SSI). At the last follow‐up, no patient had developed complications that prompted conversion to total hip arthroplasty (THA).

**Figure 4:**
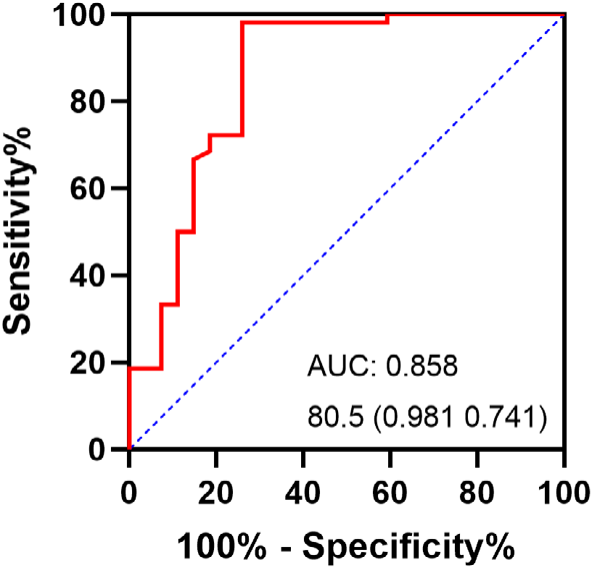
The ROC curve for the combination angle of lateral center edge angle and alpha angle. The numbers printed on the figure are summarized in the form of “threshold” (specificity, sensitivity); (AUC) the area under the curve.

In the assessment of the factors that change the PROs. Increasing preoperative alpha angle age significantly improved mHHS (*ρ* = 0.274, *P* = 0.016), HOS‐ADL (*ρ* = 0.282, *P* = 0.013), and HOS‐SSS (*ρ* = 0.364, *P* = 0.001) (Figure 4). Age, sex, BMI, joint space, and cartilage lesion degree were non‐significantly correlated with changes in PROs.

### ROC Curve Analysis

Regarding the control group, 32 hips met the inclusion and exclusion requirements. Propensity score matching yielded 27 hips in the control group and 54 in the study group. No statistical differences in age, sex, or BMI were found between the two groups. The mean LECA angle was 24.0° ± 1.3° and 23.3° ± 1.8° among the control and study groups, respectively. The alpha angle was 67.3° ± 6.7° and 55.1° ± 10.3°, respectively. The combined angle was 90.6° ± 6.8° and 79.2° ± 10.3°, respectively (Table 5).

**Table 5:** Demographic and radiographic results between the two groups

Category	Study group	Control group	*t*/*X* ^2^ value	*P*‐value
No. of hips	54	27		
Age, years	35.8 ± 10.5	37.9 ± 9.8	0.864	0.390
BMI, kg/m^2^	23.0 ± 3.4	22.4 ± 3.1	−0.895	0.374
Sex, female, *n* (%)	16 (29.6)	8 (29.6)	0.000	>0.999.
Tönnis angle	8.2 ± 3.3	7.1 ± 3.4	−1.439	0.154
LCEA angle	23.3 ± 1.8	23.7 ± 1.3	1.312	0.194
Alpha angle	67.3 ± 6.7	53.2 ± 10.0	−6.619	*P* < 0.001
Combined angle	90.6 ± 6.8	79.2 ± 10.3	−6.238	*P* < 0.001

*Note*: Values are given as mean ± SD or *n* (%).

Abbreviations: BMI, body mass index; LCEA, lateral center edge angle.

In order to identify the cut‐off value of the combined angle, the ROC curve was conducted to detect the occurrence of FAIS. Using the Youden index, the cut‐off combined angle of 80.5° was determined (AUC, 0.858; 95% CI: 0.757–0.938; Se, 98.1%; Sp, 74.1%; *P* < 0.001) for the occurrence of impingement in the BDDH population.

## Discussion

The main findings of this study were that a majority of BDDH patients can obtain clinically significant outcomes with arthroscopic surgery followed by a minimum follow‐up of 2 years. Furthermore, increasing preoperative alpha angle age was strongly associated with improving mHHS, HOS‐ADL, and HOS‐SSS (*P* = 0.016, 0.013, and 0.001, respectively). Besides, we showed that LCEA with an alpha angle of 80.5° may be a factor in predicting the incidence of FAIS in the BDDH group.

### Arthroscopy in BDDH Hips and Its Risk Factors

Although hip arthroscopy has shown some promise in treating BDDH, its efficacy in this population is still highly debated.^9^ Following hip arthroscopy, Domb et al.^21^ significantly improved clinical outcomes among BDDH patients. The revision rate was 19%. They also reported hip arthroscopy in BDDH athletes who had a minimal 2‐year follow‐up; the findings showed that the return to sport rates were 80.7%, but did not report the failure rate. Fukui et al.^22^ documented improved clinical outcome scores for 102 hips, despite a 15% failure rate. The revision rate in the current study was 3.9%. The outcomes of this study imply that BDDH patients might anticipate excellent PROs and low revision rates with cautious patient selection.

Age, sex, BMI, and joint space were unassociated with improving PROs; we attribute this to strict surgical indications. Interestingly, the increase in preoperative alpha angle was statistically associated with improving the score in mHHS, HOS‐ADL, and HOS‐SSS and was statistically uncorrelated with changes in the iHOT‐12 score. Previous studies have shown that higher alpha angles are negative predictors of clinical outcomes and independently predicted acetabular cartilage damage among patients with FAIS.^23^ However, Ouyang et al.^24^ documented that patients with FAIS with higher preoperative alpha angles tend to experience a clinically significant improvement earlier after hip arthroscopy treatment; this could be because their greater preoperative cam deformity was corrected, resulting in earlier symptomatic remission. Furthermore, we divided our patients into two groups (38 hips vs. 39 hips) according to the alpha angle of 66°. Patients with a larger alpha angel had worse preoperative mHHS, HOS‐ADL, HOS‐SSS, and iHOT‐12 scores; this may also help explain that larger alpha angle was statistically associated with net improving PROs.

### Combination of LCEA and Alpha Angles

The incidence of FAIS was influenced by acetabular coverage and femoral head shape. In BDDH patients, Ziebarth et al.^13^ observed a significant incidence of postoperative FAI signs (47.8%) following PAO in males. This might be attributed to the correction of acetabular under coverage (larger LCEA), which resulted in impingement between the femur and acetabulum. Consequently, in the setting of BDDH patients, LCEA angle is usually less than 25°, and the currently accepted cam impingement range of 50°–55° is inconvincible. Since dysplastic and impingement morphologic alterations often coexist, diagnosing impingement using a combined LCEA and alpha angles is reasonable. Combination angle 80.5° was identified as the cut‐off for detecting impingement in BDDH populations when the Youden index was used. Hayashi et al.^14^ utilized a combination of the anterior center‐edge angle and the alpha angle to detect postoperative impingement in DDH patients and determined that 108° is the threshold angle for a successful combination. Their larger combination angle was that they used the postoperative anterior center‐edge angle, which increased from 43.2° preoperatively to 62.3° postoperatively. In light of these findings, it is recommended that the assessment of impingement in BDDH hips should incorporate both the LECA and alpha measurements.

### Strengths and Limitations

This study exhibited several noteworthy strengths. Firstly, it expounded upon the efficacy of hip arthroscopy in a relatively large cohort of Asian BDDH patients with a minimum follow‐up period of 2 years. Additionally, the rate of revision was lower than that of Western populations. Secondly, the study identified a combination of LCEA and alpha angle greater than 80.5° as a dependable predictive factor for impingement in BDDH populations.

This study has certain limitations. First, there was a selection bias due to a retrospective study, especially in the control group. However, propensity score matching was used to control for potential confounding variables. The second limitation was the study's follow‐up duration, revision cases may increase with longer follow‐up, but overall revision surgery rates might still be relatively low, long‐term follow‐up study is required. Third, arthroscopy was performed on all hips in the present study. Future research should include a comparative study of arthroscopy versus PAO in patients with BDDH to evaluate the clinical outcomes of these two established treatments.

### Conclusions

Good PROs and low revision rates can be expected in the BDDH population with careful selection of patients in Asian populations. A combination angle >80.5° could be a reliable predictive factor of impingement in BDDH populations.

## Author Contributions

Fan Yang and Zheng Zhou drafted the manuscript; Hongjie Huang and Xin Zhang revised the manuscript; Xiaodong Ju and Jianquan Wang conceived the study. All authors read and approved the final draft of the manuscript.

## Funding Information

This work was supported by funds from the National Natural Science Foundation of China (Grant no. 82072403) and Innovation & Transfer Fund of Peking University Third Hospital (BYSYZHKC2021110).

## Conflict of Interest Statement

The authors have no conflict of interest to declare.

## Ethics Statement

This retrospective study was approved by the Ethics Committee of the Institutional Review Board of Peking University Third Hospital (No. M2019193). We obtained informed consent exemptions approved by the ethics committee. All methods were carried out in accordance with relevant guidelines and regulations of the Institutional Review Board.
